# Consideration of familiarity accumulated in the confined field trials for environmental risk assessment of genetically modified soybean (*Glycine max*) in Japan

**DOI:** 10.1007/s11248-020-00193-z

**Published:** 2020-01-29

**Authors:** Akane Matsushita, Hidetoshi Goto, Yasuyuki Takahashi, Mai Tsuda, Ryo Ohsawa

**Affiliations:** 1 Dupont Production Agriscience K.K., Sanno Park Tower, 2-11-1, Nagata-cho, Chiyoda-ku, Tokyo 100-6110 Japan; 2 Bayer CropScience K.K., Marunouchi Kitaguchi Bldg, 1-6-5, Marunouchi, Chiyoda-ku, Tokyo 100-8262 Japan; 3 Dow AgroSciences Japan Ltd., Sanno Park Tower, 2-11-1, Nagata-cho, Chiyoda-ku, Tokyo 100-6110 Japan; 4 Gene Research Center, Tsukuba Plant Innovation Research Center, University of Tsukuba, 1-1-1 Tennodai, Tsukuba, Ibaraki 305-8572 Japan; 5 Faculty of Life and Environmental Sciences, University of Tsukuba, 1-1-1 Tennodai, Tsukuba, Ibaraki 305-8572 Japan

**Keywords:** Genetically modified crops, Environmental risk assessment, Confined field trials, Familiarity, Data transportability

## Abstract

To date, there have been 160 regulatory approvals for environmental safety in Japan for the major genetically modified (GM) crops, including corn, soybean, canola and cotton. Confined field trials (CFTs) have been conducted in Japan for all single events, which contain various traits. The accumulated information from these previously conducted CFTs, as well as the agronomic field study data from other countries, provides a rich source of information to establish “familiarity” with the crops. This familiarity can be defined as the knowledge gained through experience over time, and used to inform the environmental risk assessments (ERA) of new GM crops in Japan. In this paper, we compiled agronomic data from the CFTs performed in Japan for 11 GM soybean events which obtained food, feed and environmental safety approvals from regulatory agencies in Japan. These CFTs were conducted by multiple developers according to Japan regulations to support the ERA of these GM soybean, covering standard measurement endpoints evaluated across developers in Japan. With this dataset, we demonstrate how familiarity gained from the CFTs of GM soybeans in Japan can be used to inform on the ERA of new GM soybean events. By leveraging this concept of familiarity, we discuss potential enhancements to the ERA process for GM soybean events in Japan.

**Electronic supplementary material:**

The online version of this article (10.1007/s11248-020-00193-z) contains supplementary material, which is available to authorized users.

## Introduction

Japan is one of the top import countries of genetically modified (GM) crop products, especially from the US (USDA 2013). Although no GM crops are commercially cultivated in Japan, both cultivation approval and food and feed safety approvals are required for import and use of GM crops as food, feed and processing. The cultivation approval assesses the potential environmental impacts if, for example, imported grain is spilled during transportation or if conventional seeds that have been imported for planting in Japan are contaminated with GM events. As part of the environmental risk assessment (ERA), confined field trials (CFTs) are required to be conducted in Japan, to compare the agronomic and phenotypic characteristics between the GM crop and an appropriate non-GM near isogenic control variety. The requirements for ERA, as a part of the GM product assessment and authorization, are specified by two regulatory agencies, the Ministry of Agriculture, Forestry and Fisheries (MAFF) and the Ministry of Environment (MOE), under the domestic law enforced in 2004 to implement the Cartagena Protocol on Biosafety (J-BCH 2018a). In 2004, when the current regulatory framework was established, the requirement to generate in-country CFT data to inform the ERA for import was likely posed to address uncertainty about the potential effects of the Japanese environment on phenotypic and agronomic characteristics of GM crops (Yogo 2010). In comparison, Environmental Risk Assessments (ERA) and in-country CFTs are not typically required for import approvals in other countries (EFSA 2010; RDA 2014). This existing requirement for conducing CFTs in Japan should be reassessed to determine if it informs the ERA.

There is a two-step approval process that must be followed, prior to submitting an application for cultivation approval in Japan: first, an ERA for CFT use is done considering laboratory and glass-house data (to characterize the intended trait, including molecular characterization studies and bioassays) and field data which is collected in other countries, typically in US. Next, if CFT approval is granted, GM events are planted along with non-GM controls in CFTs in Japan, to allow a comparative assessment of plant characteristics, e.g., phenotypic and agronomic characteristics between the GM crop and an appropriate non-GM near isogenic control variety. Using this information, in addition to host plant information and other related information, the following three assessment endpoints are evaluated: (1) competitiveness of the GM plant with wild plants for resources such as nutrients, sunlight, habitat, etc.; (2) production of harmful substances that interfere with the growth of wild plants, animals, or microorganisms; and (3) outcrossing potential with wild relatives, resulting in introgression of transgenic DNA (J-BCH 2018b). If one or more unanticipated (other than the intended trait) statistically significant differences are observed between the GM crop and the control in the CFT, the differences need to be assessed for biological relevance, specifically assessing if the GM plant will have increased competitiveness with wild plants, will produce harmful substances that affect wild plants, animals, or microorganisms, or will have increased outcrossing potential. To assess biological relevance of an observed difference, it is important to understand the basic biology of the crop, the characteristics of the introduced trait, the receiving environment for the GM crop, and the potential interactions between these factors (Nickson 2008). Biological relevance of observed difference can be evaluated by considering if the values for the GM crop fall within the range of the values for non-GM crops described in literature. One limitation of Japan’s required CFT is that publicly available agronomic data from GM and its near isogenic line of non-GM crops grown in Japan is currently not available to assist this assessment, which makes understanding biological relevance difficult.

Environmental risk assessment should be conducted using problem formulation, following a science based approach, and leveraging familiarity. A problem formulation based approach to risk assessment assesses existing data to determine if there is a plausible and testable hypothesis for environmental harm. This problem formulation approach helps identify if additional data is needed to inform the risk assessment (Raybould and Macdonald 2018). Additionally, the globally recognized concept of familiarity was originally developed by OECD (1993) and is another key concept for the risk assessment of GM crops. Familiarity can be defined as the knowledge gained through experience over time. The concepts of both problem formulation and familiarity should be considered when determining if CFT conducted in Japan will inform the risk assessment. For example, large multi-location field trials, which are carried out typically in the country of development, are performed and the comparative assessments of the agronomic and phenotypic characteristics are used to support the ERA for cultivation of GM crops (Garcia-Alonso et al. 2014). However, in the case of the Japan CFTs, the null hypotheses of no difference between a GM crop and a comparator is tested in the Japanese environment, even if data from another geography demonstrates no difference and there is no plausible hypothesis for how the event would be different in the Japanese environment. To date, there have been around 160 regulatory approvals for environmental safety in Japan for the major GM crops, including corn [*Zea mays* subsp. *mays* (L.) Iltis], soybean [*Glycine max* (L.) Merr.], canola (*Brassica napus* L.) and cotton (*Gossypium hirsutum* L.), and CFTs have been conducted in Japan for all single events. Assessed events have expressed various traits, including herbicide tolerance, insect resistance and nutritional improvement. Based on the CFT data conducted in Japan, the ERA for all of these events concluded that the GM crop exhibits no adverse effect upon the natural environment in Japan and are comparable to conventional crops, with the exception of the introduced trait. In all cases to date, the conclusions from the CFTs in Japan are the same as the conclusions from agronomic data developed from cultivation (exporting) countries. Therefore, the agronomic data collected to support cultivation approvals in other countries can be leveraged for the ERA in Japan, and a CFT in Japan should only be required if there is a plausible hypothesis for how GM event would lead to adverse effects in the Japanese environment.

Recently, the regulatory authorities in Japan have updated the regulations for GM maize containing familiar traits. In cases where the GM maize contains a transgene that has previously been reviewed in Japan (e.g., MAFF 2014), the ERA can be conducted using CFT data from other countries. The Japanese regulators recognized that the accumulated information from previously conducted CFTs in Japan, as well as the agronomic field study data from other countries, provides a robust data set, and the resulting familiarity can be used to inform the ERA of new maize events containing similar traits in the future. To date, there have been three GM corn events which satisfied these criteria and were exempted from in-country CFTs. On the other hand, for other crops like soybean, an in-country CFT is still required to be conducted for the ERA in Japan.

In this publication, we compiled agronomic data from eleven CFTs of GM soybean events, which can be used to increase familiarity and improve the evaluation system for ERA of GM soybean in Japan. These CFTs have been conducted by multiple developers to support product approval for environmental safety in Japan, but the data has not been disclosed previously in the literature. Based on the compiled data which covers common measurement endpoints currently evaluated in the CFTs, we demonstrate how familiarity gained from the data accumulated in the CFTs of GM soybean in Japan can be used to inform on the ERA of new GM soybean events. Furthermore, leveraging the concept of familiarity, we discuss potential enhancements to the ERA process for GM soybean in Japan including the transportability of CFT data between countries and leveraging existing data to inform the risk assessment of new events.

## Materials and methods

### Events

Since 2004, a total of sixteen GM soybean events have been evaluated after implementation of the Cartagena domestic law in Japan. The developers of each soybean event were consulted to determine if the CFT data from Japan could be released for use in this publication. Of these sixteen soybean events, the CFT data from eleven events were approved for use in this publication by Monsanto (now Bayer CropScience), Dow AgroSciences and Dupont Pioneer (now Corteva Agriscience). These events are summarized in Table 1, including herbicide tolerance (HT), insect resistance (IR) and nutritional improvement (NI) traits. In CFTs, each event was compared with a corresponding near-isogenic control line (NIL).

**Table 1:** Eleven GM soybean events that were tested in CFT in Japan and authorized in Japan since 2004

OECD UI	Developer	Trait				Inserted gene(s)
		HT	IR	NI	Detail	
DAS-68416-4	Dow AgroSciences	✓			Aryloxyalcanoate and glufosinate tolerance	*aad*-*12*, *pat*
DAS-444Ø6-6	Dow AgroSciences	✓			Aryloxyalcanoate, glyphosate and glufosinate tolerance	*aad*-*12*, *2mepsps*, *pat*
DAS-81419-2	Dow AgroSciences	✓	✓		Lepidopteran resistance and glufosinate tolerance	*cry1Ac*, *cry1F*, *pat*
DP-356Ø43-5	Dupont Pioneer	✓			Glyphosate and sulfonylurea tolerance	*gat4601*, *gm*-*hra*
DP-3Ø5423-1	Dupont Pioneer	✓		✓	High oleic acid and sulfonylurea tolerance	*gm*-*fad2*-*1*, *gm*-*hra*
MON-89788-1	Monsanto Company	✓			Glyphosate tolerance	*cp4epsps*
MON-87769-7	Monsanto Company			✓	Stearidonic acid production	*Pj*.*D6D*, *Nc*.*Fad3*
MON-877Ø1-2	Monsanto Company		✓		Lepidopteran resistance	*cry1Ac*
MON-877Ø5-6	Monsanto Company	✓		✓	Low saturated fatty acids, high oleic acid and glyphosate tolerance	*fad2*-*1A*, *fatb1*-*A*, *cp4epsps*
MON-877Ø8-9	Monsanto Company	✓			Dicamba tolerance	*dmo*
MON-87751-7	Monsanto Company		✓		Lepidopteran resistance	*cry1A*.*105*, *cry2Ab2*

*HT* herbicide tolerance, *IR* insect resistance, *NI* nutritional improvement

### Overview of CFT experimental design

Each CFT was conducted independently by the corresponding developers from 2005 to 2015 in confined fields that are located across Japan, including Ibaraki, Tochigi, Shizuoka and Fukuoka (Online Resource 1). Cold tolerance of some events were measured in the glasshouse located in US, see Supplementary information). Cultivation was performed under conventional field management practices. All studies except outcrossing rate were comparative studies, where the GM crop and the non-GM NIL comparator were grown in the same field, and their growth and agronomic characteristics were compared, based on the evaluation items indicated in the guideline (J-BCH 2018a). The experimental protocols used by each developer are similar, but not identical; for example, the number of samples or the number of replicates may be different, depending on the protocol used by the developer. Hence, hereafter generalized methods that were used for each evaluation item are described. For more detail, see Online Resource 2. Likewise, the methods for statistical analysis adopted by each developer are similar, but not identical. Statistical details for each CFT are not provided here, but the appropriateness of the statistical methods used for each CFT were reviewed and confirmed by academic experts during regulatory review processes.

### Agronomic characterization

Measurement endpoints for the agronomic characteristics that are commonly used by developers in CFTs for ERA in Japan are; germination rate, flowering stage, maturity stage, main stem length, number of nodes, number of branches, number of pods, pod shattering, weight of grain and weight of 100 grains. Definition of each endpoint is provided in Online Resource 3.

### Cold tolerance during the early stages of growth

Seeds were planted in pots and grown in a glasshouse or a growth chamber (20–25 °C). Seedlings at the V1 growth stage were transferred to either a growth chamber where the average temperature was maintained at approximately 4 °C or an open plot where the average temperature was 3 °C. Survival or damage (wilting, fading and necrosis) of the seedlings was either monitored by visual observation or by assessing plant height and dry weight, which were measured after 2–4 weeks to determine any differences between the GM and NIL plants in terms of their tolerance to cold temperatures.

### Overwintering

After the harvest period, unharvested plants were left on the trial field and were monitored to determine if they survived through the following winter period. Plant survival was monitored until the end of the trial (Online Resource 1), to determine any differences between the GM and NIL plants in terms of their ability to survive over the winter.

### Pollen viability observation

Unopened flower buds were dissected from plants at the flowering stage. Anthers within were removed and dipped into either 1–3% potassium iodide solution, Alexander solution (Alexander 1969), or acetocarmine solution. Subsequently, a sample of the solution which contained soybean pollen was removed and observed under an optical microscope. The ratio of stained pollen grains to total pollen grains was observed, and the diameter of pollen grains was measured to determine any differences between the GM and NIL plants in terms of their pollen viability.

### Germination rate and dormancy of harvested seeds

At harvest, seeds were collected from 15 to 48 plants in each plot and seeds were bulked. Seeds from each bulked sample were sown in an open plot or in culture dishes without any treatment to assess germination rate and seed dormancy. At least 5 days after planting, germination rates were determined by calculating the ratio of the number of established seedlings in the open plot or the number of germinated seeds in culture dishes to the total number of seeds used for the study.

### Succeeding crop test

At harvest, soil samples (approximately 20 × 20 × 20 cm^3^) were collected from underneath several soybean plants in each plot. Root tissues were removed, and the soil samples were composited for each plot. Field soil from each plot was then placed in pots and radish seeds were planted and maintained at 25 °C in a controlled environment. After 1–2 weeks, the seedling establishment rate for radish seeds was measured. Additionally, soil from representative seedlings was gently removed, and after drying seedlings in a thermal incubator, the dry weight of the seedlings was measured.

### Plough-in test

Soybean plants were harvested from the field and were dried in a controlled incubator or glasshouse. The dried plant materials were ground with a grinder mill, and the powdered materials were pooled and incorporated into potting media at a rate of 0.5 or 1% to make the test soil. Test soil was placed in pots and radish seeds were planted and maintained at 25 °C in the controlled environment. After 1–2 weeks, the seedling establishment rate for radish seeds was measured. Additionally, soil from representative seedlings was gently removed, and after drying seedlings in a thermal incubator, the dry weight of the seedlings was measured.

### Soil microflora test

Soil samples (approximately 400 ml) were taken from underneath plants in each field plot and soil microorganisms were counted using a dilution plate method (Asanuma et al. 2011). A small sub-sample of each soil sample was mixed with sterilized water or an appropriate buffer. Supernatant was removed to create the first diluted solution (10^−1^), and then tenfold serial dilutions were prepared. An aliquot of each dilution was inoculated onto Rose Bengal agar medium for filamentous fungi, and PTYG agar or similar medium for bacteria and actinomycetes, and then plates were incubated in the dark at 25 °C for 7 days. Several replicate plates of each media and dilution were used, and plates containing the optimal density of colonies (10–300 colony forming units; CFUs) were counted. The total number of bacterial, actinomycete and filamentous fungal CFUs were calculated and reported as the number of CFUs per 1 g of dry soil.

### Outcrossing test

Non-GM NIL plants were planted in the field site in an area adjacent to GM plants and both plants were grown to maturity. To assess outcrossing from GM plants to non-GM NIL plants, seeds were collected from the non-GM NIL plants and were subsequently planted and grown to the V2–V4 stage in an open plot or in pots and grown in a glasshouse. To determine if outcrossing occurred (i.e., presence of a GM trait in the non-GM NIL progeny), the progenies of the non-GM plants were either sprayed with herbicide (the herbicide that corresponds to the herbicide resistance trait in the GM plants), characterized by PCR (to detect DNA sequence of the transgene), or assessed by lateral flow immunochromatographic assays (to detect the transgenic protein).

## Results: dataset compiled from CFTs for eleven soybean events

### Agronomic characterization

The common agronomic characteristics measured in CFTs across the 11 soybean events include germination or seedling establishment rate (G/SE rate; %), flowering stage (date), maturity stage (date), main stem length (cm), number of nodes, number of branches, number of pods, and pod shattering (J-BCH 2018c; Table 2). With the exception of two soybean events, DAS-68416-4 (99.0% for GM vs. 80.2% for non-GM NIL) and MON-877Ø8-9 (99.2% for GM vs. 100% for non-GM NIL), there were no statistically significant differences in G/SE rate between the GM and non-GM NIL plants across the CFTs (Table 2). The G/SE rate of DAS-68416-4 soybean was reevaluated in the glass house, and exhibited no statistically significant differences in this assessment (98.3% for GM vs. 95.0% for non-GM NIL), indicating that the statistically significant difference observed in seedling establishment rate in the field for this event may not have been a true biological difference. Similarly, while the G/SE rate of MON-877Ø8-9 was decreased compared to control, the rate of MON-877Ø8-9 remained above 99%, indicating that this difference is not biologically relevant. With the exception of MON-87751-7 (104.2 cm for GM vs. 108.7 cm for non-GM NIL), there were no statistically significant differences between GM and corresponding non-GM NIL plants for main stem length (Table 2). In this case, the main stem length of MON-87751-7 was determined to fall within the range of values reported for other commercial varieties (J-BCH 2018c), indicating this difference is not biologically relevant. With the exception of MON-877Ø5-6 (7.0 for GM vs. 6.1 for non-GM), there were no statistically significant differences between GM and corresponding control for number of branches (Table 2), and no statistical differences were observed for any soybean event for number of nodes (Table 2) or number of pods (Table 3). Since there was no significant impact on the number of pods per plant in MON-877Ø5-6 soybean, there is no evidence of changing seed production ability.

**Table 2:** Agronomic characteristics of GM and non-GM control soybeans tested in CFTs in Japan

OECD UI	G/SE rate (%)		Flowering time (MM/DD)		Maturity date (MM/DD)		Main stem length (cm)		Number of nodes		Number of branches	
	GM	Control	GM	Control	GM	Control	GM	Control	GM	Control	GM	Control
DAS-68416-4	99.0* (98.3)^a^	80.2 (95.0)	8/6–9/15	8/6–9/15	10/20	10/20	84.8	86.5	23.1	23	7.8	7.9
DAS-444Ø6-6	94.3	86.5	8/6–9/15	8/6–9/15	10/20	10/20	97.6	89.3	23.8	23	9.2	8.2
DAS-81419-2	92.2	94.8	8/26–10/2	8/26–10/2	11/1	11/1	52.0	62.9	15.9	15.6	4.0	4.0
DP-356Ø43-5	87	89	8/1	8/1	10/16	10/16	47.5	55.2	13	13.7	1.9	2.3
DP-3Ø5423-1	99.3	97.2	7/9	7/8	10/23	10/23	61.8	59.3	14.3	14.3	4.2	4
MON-89788-1	99.17	97.78	7/27–9/8	7/27–9/8	10/17	10/17	67.63	67.97	18.40	18.53	7.60	7.80
MON-87769-7	96.72	97.97	8/21–9/16	8/21–9/16	11/10	11/10	68.13	68.11	18.58	18.88	5.73	5.68
MON-877Ø1-2	71.3	76.0	9/4–9/24	9/4–9/24	12/8	12/8	59.0	56.2	13.6	13.5	7.0	6.5
MON-877Ø5-6	98.3	91.6	8/2–9/2	8/2–9/2	11/9	11/9	71.1	73.5	19.6	20.5	7.0*	6.1
MON-877Ø8-9	99.2*	100.0	7/16–9/8	7/16–9/8	11/2	11/2	82.1	78.5	21.1	21.0	7.3	7.4
MON-87751-7	NC^b^	NC	7/8–8/12	7/4–8/12	10/29	10/29	104.2*	108.7	21.9	22.4	6.7	6.8

Average values for each measurement endpoints related to agronomic performance are provided. Since each value was taken from original dossiers as is, significant figures are not consistent. Asterisk indicates statistically significant difference (*P* < 0.05)

^a^G/SE rate of DAS-68416-4 was reevaluated in the glass house and exhibited no statistically significant difference between GM and non-GM NIL (98.3 vs. 95.0%, respectively)

^b^*NC* not conducted

**Table 3:** Productivity of grain and germination rate of harvested seeds of the GM soybeans

Event name	Number of pods^a^		Grain weight per plant (g)^a^		Weight of 100 grains (g)^a^		G/SE rate for harvested seeds (%)^a^	
	GM	Control	GM	Control	GM	Control	GM	Control
DAS-68416-4	199.1	223.4	69.9	85.1	15.2	15.5	100	100
DAS-444Ø6-6	269.2	289.2	102.8	120.4	16.1	16.2	100	100
DAS-81419-2	83.6	95.8	30.5	39.1	16.9	17.9	100	100
DP-356Ø43-5	43.9	57.1	16.7	20.4	16.9	15.7	97	100
DP-3Ø5423-1	72.6	73.5	33.3	31.6	20	19.2	98.5	98
MON-89788-1	138.13	134.80	52.87	57.82	17.12	18.54	100.00	100.00
MON-87769-7	92.45	94.48	38.71	39.38	20.33	21.58	99.44	100
MON-877Ø1-2	67.1	69.1	31.2	29.8	25.3	24.9	91.7	94.4
MON-877Ø5-6	105.6	103.1	45.3	47.6	18.2*	19.4	98.9	98.9
MON-877Ø8-9	122.7	125.8	54.7*	51.0	20.6*	19.6	98.5	98.0
MON-87751-7	133.4	134.9	64.9*	58.8	24.0	24.9	98.5	97.0

^a^Average values for each measurement endpoint are provided. Since each value was taken from original dossiers without modification, significant figures reported are not consistent. Asterisk indicates statistically significant difference (*P* < 0.05)

Flowering time and maturity date endpoints were not statistically analyzed, and all GM and non-GM NIL controls had similar dates for both endpoints. Only two soybean events had slightly different flowering times (DP-3Ø5423-1 and MON-87751-7), however these differences were very small (with only a few days of difference; Table 2). Results for pod shattering across 11 CFTs were consistent, where there were no reported differences in pod shattering between the GM and corresponding non-GM NIL controls based on visual observation of representative plants.

### Cold tolerance of early developmental stage and overwintering

To assess cold tolerance, the health and viability of GM and non-GM NIL soybean plants were assessed after being grown under cold temperatures in growth chamber (4 °C) or open plot. Across all 11 CFTs, all GM and non-GM NIL withered after growing for 2–5 weeks under these low temperature conditions. No differences were reported between any of the GM soybean events, when compared to their respective non-GM NIL controls, indicating that these GM traits did not increase the tolerance of the GM plants to cold. Similarly, when GM plants were compared to non-GM NIL control plants, there were no differences in their ability to survive the winter season. All plants, whether they were GM or non-GM soybean, withered and did not survive the overwintering test.

### Pollen viability and size

After staining, the pollen from GM and non-GM NIL plants was observed microscopically to determine viability. There were no statistically significant differences in pollen viability (staining %) of the GM soybean events and their respective non-GM NIL controls for any of the events analyzed quantitatively (Online Resource 4). There were also no statistical differences in pollen size between GM soybean events and their respective non-GM NIL controls for any of the events analyzed quantitatively (Online Resource 4). Some protocols used by some of developers did not assess pollen viability using a quantitative method, however no visual differences in pollen viability were reported for these events, and all GM soybean plants across the 11 CFTs were reported to have viable and normal-shaped pollen.

### Productivity of grain

Across the 11 CFTs, total number of pods, grain weight per plant, weight of 100 grains and germination of harvested seeds were measured (J-BCH 2018c; Table 3). Statistically significant differences were observed in grain weight per plant and the weight of 100 grains for MON-877Ø8-9 compared to the controls (54.7 vs. 51.0 g and 20.6 vs. 19.6 g, respectively; Table 3), The differences in weight of 100 grains for MON-877Ø5-6 compared to the control (18.2 g vs. 19.4 g; Table 3) and in grain weight per plant for MON-87751-7 (64.9 g vs. 58.8 g; Table 3) were also statistically significant.

### Germination or seedling establishment rate for harvested seeds

The G/SE rate of harvested seeds from both GM soybean and the non-GM NIL controls were measured by collecting seeds at harvest and replanting them in open plots or culture dishes (J-BCH 2018c). For all events across the 11 CTFs, the GM soybean and the non-GM NIL control soybeans both germinated or established at a similar rate, with rates exceeding 90% (Table 3).

### Succeeding crop and plough-in tests

To assess the potential effects of the GM soybean plants on a succeeding crop, radish seeds were planted in soil from the GM or non-GM soybean field plots (succeeding crop test) or were planted in media containing ground GM or non-GM soybean plant tissue (plough-in test). In both tests, there were no statistically significant differences observed in the G/SE rate or dry weight of radish plants that were grown under either assessment (Online Resource 5), indicating that the GM soybeans did not affect the ability of the succeeding crop to germinate or grow.

### Miscroflora test

No statistically significant differences were observed in the number of micro-organisms (bacteria, actinomycetes and filamentous fungi) in soil collected from GM soybean plants, compared to soil collected from their non-GM controls (Online Resource 5).

### Outcrossing test

Outcrossing potential was determined using GM soybean as pollen donor and assessing soybean seeds obtained from non-GM control (planted adjacent to GM soybean plots) to determine if any of the hybrid progeny seeds contained the transgene. The outcrossing rate across all 11 GM soybean events was consistent and low, where outcrossing from most of the events was 0%. The few events that were above 0% had outcrossing rates of 0.10–0.23% (Online Resource 6), which are still within the range of natural outcrossing rates reported in literature.

### Additional analysis of agronomic data from CFTs: correlation analysis

**Fig. 1:**
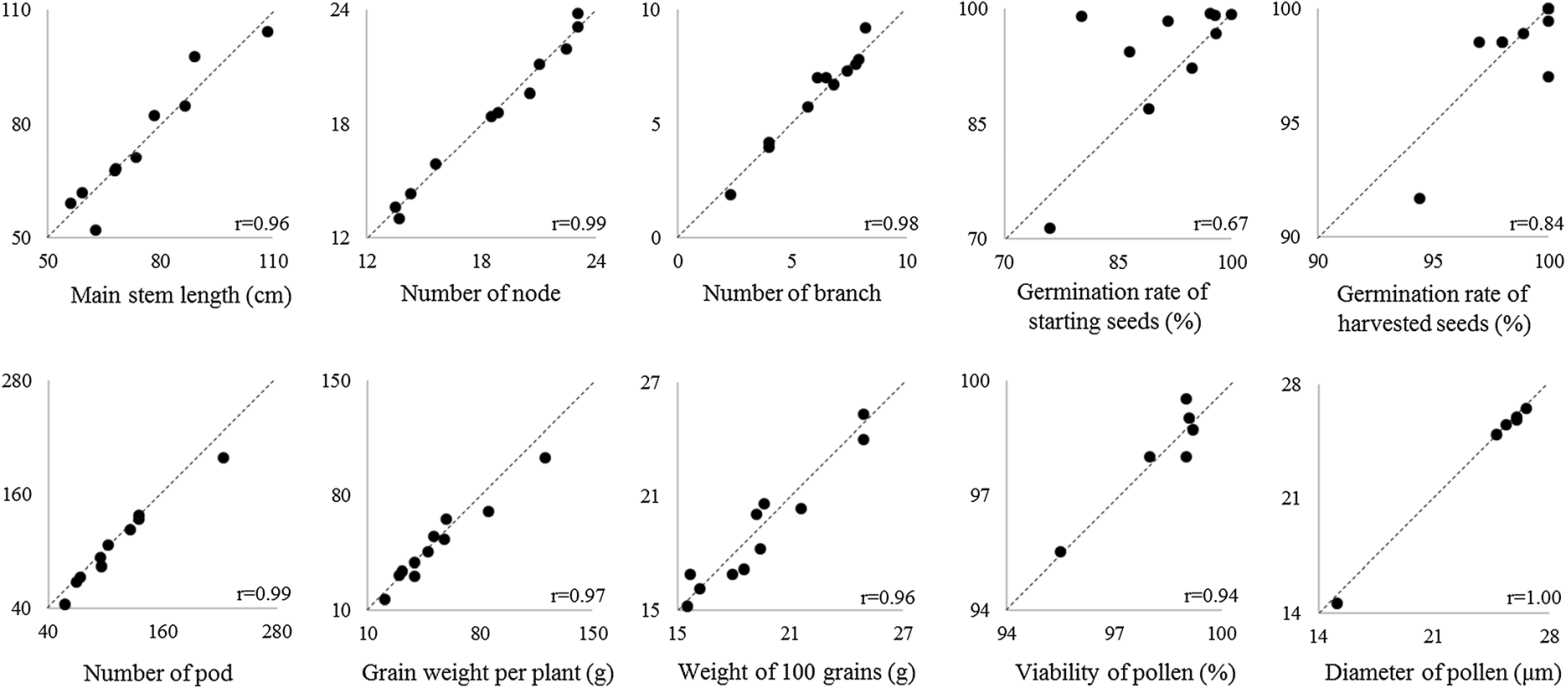
Correlation in key agronomic characteristics between GM soybean and non-GM comparator in Japan CFT. Each pair of the mean value of the major measurement endpoints for the 11 GM soybean (*Y* axis) and corresponding non-GM NIL soybean (*X* axis) were plotted, and Pearson’s r was calculated by dividing the covariance of X and Y by the standard deviations of X and Y. Dotted lines in each chart represent Y = X

To better visualize any differences between GM soybeans and non-GM soybeans and to identify any potential unintended impacts, the agronomic endpoints related to competitiveness were also compared using two-dimensional correlation analysis (Fig. 1). A range of variation was observed in the mean value of each measurement endpoint for non-GM soybeans (*X* axis), which is also the case for GM soybeans (*Y* axis). The mean values between each GM soybean and its corresponding non-GM soybean control are not necessarily equal, but the individual data points representing each pair are distributed around Y = X and a strong correlation was observed in most of the measurement endpoint (R ≥ 0.80). Additionally, no statistically significant differences were observed between GM soybean and non-GM control soybean for these endpoints. The only low correlation was observed in germination rate of starting seed. For this endpoint, the difference in the conditions used for storage or treatment of seeds used for each CFT likely caused the variation observed here for germination rate of starting seed. Thus, it is unlikely that the difference in the germination rate of starting seeds is biologically relevant.

## Discussion

The ERA of GM crops in Japan aims to test the hypothesis that the GM crop is not different than the conventional crop in terms of its potential impact on domestic biodiversity by evaluating competitiveness, production of harmful substances, and ability to outcross. Other countries, with the exception of China, do not require an ERA with in-country CFTs for import of grain for food and feed use. Even though no cultivation is intended in Japan, in-country CFTs are required for the ERA of GM crops to assess the potential for environmental impacts if the GM grain is unintentionally grown (for example, if grain is spilled during transportation, at port of entry). This paper has been prepared with two goals in mind: First, to publish a comprehensive dataset of soybean agronomic data from GM and non-GM soybean grown in CFTs in Japan; and second, to question the value of requiring in-country CFTs for cultivation approval in Japan.

### Comprehensive dataset of agronomic data for soybeans grown in Japan environment

Any statistically significant differences detected in the CFT need to be assessed to determine potential for biological relevance to above mentioned three assessment endpoints. Leveraging familiarity (for example, understanding the nature of the crop and the characteristics of GM traits) and existing data is very important for being able to assess results of CFTs and objectively assess biological relevance of difference observed in the receiving environment of Japan. However, a limitation of CFT is that agronomic data from GM crops and their near isogenic line of non-GM crops grown in Japan is currently not publicly available to assist the assessment, which makes understanding biological relevance difficult. Therefore, one goal of this paper was to summarize and publish a comprehensive dataset from CFTs for GM soybean previously conducted in Japan, which will build familiarity and inform the biological relevance assessment for future soybean CFTs.

In this study, CFT data from 11 GM soybean events were collected and summarized. In each CFT, a comparative assessment of the agronomic characteristics was conducted to determine differences between the GM crop and a near-isogenic comparator line which were both grown under representative environmental conditions and management practices in Japan. The datasets from each CFT were used to evaluate three assessment endpoints: (1) competitiveness of the GM plant with wild plants for resources such as nutrients, sunlight, habitat, etc.; (2) production of harmful substance that interfere with the growth of wild plants, animals, or microorganisms; and (3) outcrossing potential with wild relatives, resulting in introgression of transgenic DNA. Specifically, a suite of agronomic measurement endpoints (summarized in Tables 2, 3, and Online Resource 4) were used to assess if the GM plant has increased competitiveness. The measurement endpoints in succeeding crop test, plow-in test and microflora test (Online Resource 5) were used to assess if the GM crop interferes with the growth of other plants, animals, or microbes, and the measurement endpoints in outcrossing test (Online Resource 6) were used to assess potential for outcrossing with wild relatives.

Results from all 11 CFTs conducted in Japan for different GM soybean events reported no adverse effects on these three assessment endpoints (J-BCH 2018c). Where measurement endpoints measured across all these comparisons reported statistically significant differences between GM soybean and non-GM comparators for some events (for example, G/SE rate, main stem length, and number of nodes; Table 2 and grain weight per plant and weight of 100 grains; Table 3), a biological relevance assessment was conducted.

All of the CFTs and the biological relevance assessments were conducted independently by each of the developers of the GM products. Although the criteria adopted across these 11 CFTs were not identical due to differences in field study methods between developers, all these measurement endpoints were determined to be comparable between GM crop and non-GM control, in a case-by-case manner. This accumulated agronomic data from CFTs of GM soybean can be used to build familiarity and inform the biological relevance assessment of future CFTs for GM soybeans grown in Japan. Also, the ERA of these GM soybeans can be used to inform on the ERA for future GM soybean events.

### Informing the biological relevance assessment

It is important to understand the natural variability in agronomic endpoints for soybeans grown in Japan. Irrespective of the introduced trait, there is expected to be variability in measurement endpoints for different agronomic parameters. This can be observed in the data presented in this study for non-GM NIL soybeans across the 11 CFTs. The observed natural variation in the mean value for each agronomic characteristic of non-GM soybeans summarized from these CFTs (Tables 1, 2 and Online Resource 4–6) can serve as a reference to inform on the biological relevance assessment of soybeans in future CTFs conducted in Japan. Agronomic characteristic between GM soybean and the non-GM comparator could be statistically different in CFTs due to genetic and environmental variation, as demonstrated in several examples above; however, the variation in agronomic characteristics of host crops grown in the test fields can serve as a clear baseline to interpret biological relevance of the difference identified between GM soybean and the control. Although the differences in the methods across developers for certain measurements needs to be considered (see Supplementary information for more detail), the dataset of non-GM soybean shown here was obtained under the common objective of CFTs and can be referred to for biological relevance assessments in the future. While it could be argued that there is low genotypic diversity in the non-GM soybeans used across these eleven CFTs (Jack, Maverick, A3244, A3525, A5547, A3555), there is considerable environmental variation represented (9 different environments in terms of year × site; Online Resource 1). Thus, these data represent various conditions in Japan, and the dataset can be added to over time as new data is available to increase genotypic and environmental diversity.

### Informing the ERA for future GM soybean events

The goal of the CFT for these GM soybean events is to identify any unintended changes from the transformation process or the introduced traits on the phenotypically observable agronomic characteristics of the GM soybean. As shown using correlation analysis, there are no biologically meaningful difference between GM soybeans and non-GM soybeans for agronomic endpoints related to competitiveness (Fig. 1). Moreover, the differences between GM and corresponding non-GM soybeans (indicated by the distance of each data point from Y = X; Fig. 1) are much smaller than the differences within the non-GM soybeans (indicated by the range of X in the measured agronomic endpoints; Fig. 1), indicating that the natural variation within non-GM soybeans results in greater differences in these agronomic endpoints than that introduced with the genetic modification.

The information from the eleven independent CFTs of GM soybean summarized here supports that the agronomic characteristic of GM soybean is comparable to their respective non-GM NIL soybean comparators. This information can be used to inform the ERA for future GM soybean events, since no biologically relevant differences that could lead to environmental harm in the Japanese environment were observed irrespective of traits conferred by the inserted genes.

### Assessing the value of requiring in-country CFTs for cultivation approval in Japan

#### Familiarity of the crop

The basic biology of cultivated soybean is well documented in the literature and can be leveraged for familiarity of safe use in Japan. Soybean is commonly considered one of the oldest cultivated crops (Hymowitz 1970) and has long history of safe use in Japan. Soybean plants are annuals and do not survive vegetatively in geographic regions with cold winters from one growing season to the next as soybean seeds are the only survival structures (Hymowitz and Singh 1987). Since soybean seeds do not retain high germination rates and vigor for long periods, fresh, properly grown and handled seed is required for commercial varieties each growing season (TeKrony 1987). Conventional soybean does not possess any of the attributes commonly associated with weeds, such as long persistence of seed in the soil, the ability to invade and become a dominant species in new or diverse landscapes, or the ability to compete well with native vegetation (Baker 1974), and is known to have low weediness potential (OECD 2000).

#### Familiarity with GM soybeans

In the ERA for a GM soybean event in Japan, intended trait should be assessed to determine if any plausible hypotheses can be developed for how the GM soybean could have increased weediness in Japanese environment. To date, no plausible hypotheses have been developed for ERA of GM soybean (for example, no changes in agronomic characteristics related to weediness were observed in field studies conducted in other countries), and the conclusions that GM soybeans are as safe as non-GM soybeans is supported by decades of safe use. CFTs were originally required to address concerns that unanticipated phenotypic or agronomic changes in GM crops could occur in the Japanese environment. However, the results of these comprehensive CFTs done in Japan, which conclude that there are no biologically relevant unintended changes in agronomic characteristics, supports a conclusion that unintended changes in GM soybean are unlikely in the Japanese environment. In fact, the likelihood of having any biologically relevant differences in the GM plant that could lead to environmental harm is remote, due to the extensive breeding, screening, and advancement cycles that GM crops, like non-GM crops, advance through as part of the conventional breeding process and for commercialization (Glenn et al. 2017; Prado et al. 2014). In this breeding program, events with any unfavorable characteristics which would limit commercial viability are eliminated and not advanced through the breeding and screening process.

#### Familiarity based on existing agronomic data from other countries

GM crops are extensively tested for specified criteria (e.g., molecular characterization, phenotypic and agronomic performance; Glenn et al. 2017; Prado et al. 2014) in the country where the GM crop was developed to collect data to support global regulatory approvals. The data package submitted to regulatory authorities usually includes agronomic characterization of GM crops collected from large multi-location field trials. Prior to conducting the Japan CFTs, the 11 GM soybean events assessed in this paper were evaluated in multi-location field trials in the US and other countries, which provides agronomic data from broad range of environmental conditions and agricultural ecosystems representative of where the crop is typically grown. Furthermore, these soybean lines all have been tested and approved for environmental safety for growing (not only for import as in Japan) in the US and Canada, and many have also been approved for growing in Brazil and/or Argentina. The extensive agronomic datasets from these multi-location field studies in other countries should be transportable and used to inform the ERA for cultivation approval in Japan. This is especially true considering that CFTs in Japan are conducted at a single site in single year (Nakai et al. 2015).

**Fig. 2:**
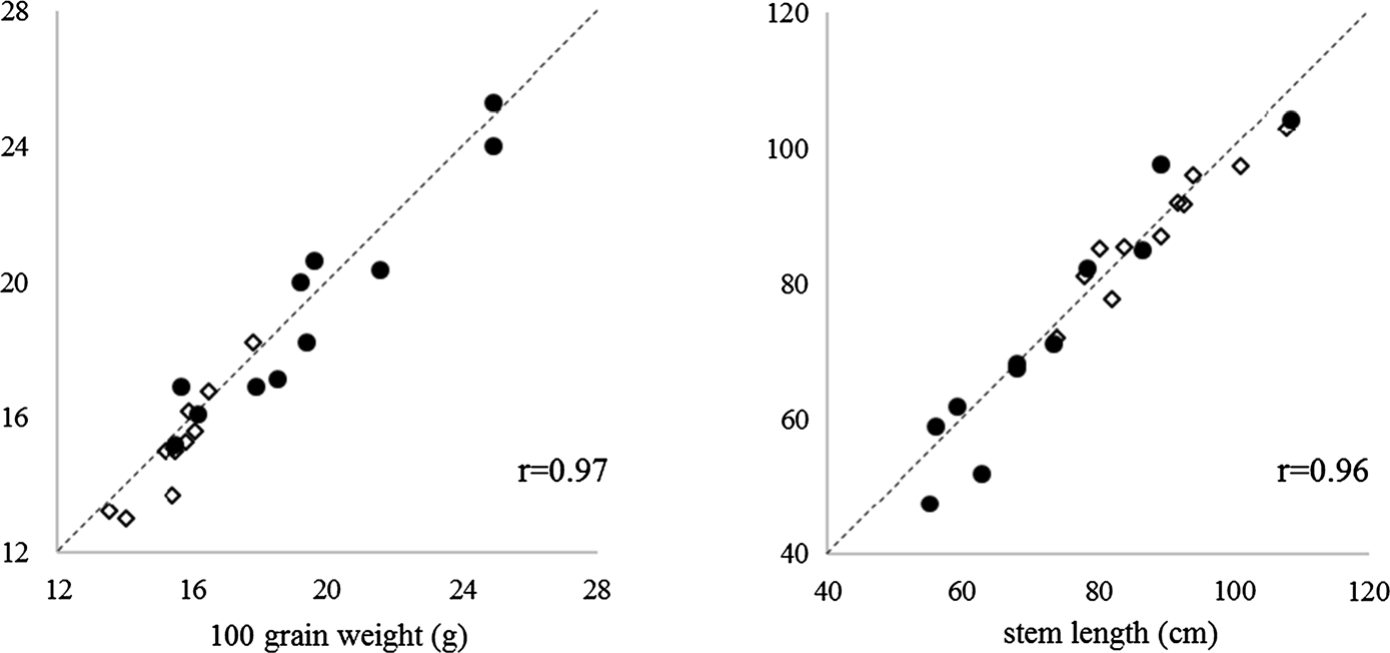
Distribution of 100 grain weight and stem length data collected for GM soybean and non-GM comparator in Japan and US CFTs. Each pair of the mean value of the two common measurement endpoints between Japan CFT (circle) and in US CFTs (diamond) for the 11 GM soybean (*Y* axis) and corresponding non-GM soybean (*X* axis) were plotted, and Pearson’s r was calculated by dividing the covariance of X and Y by the standard deviations of X and Y. Dot lines in each chart represent Y = X

To demonstrate that the dataset obtained from CFTs in Japan and multi-location field studies from the US are similar in terms of results, we compared the datasets from Japan and the US CFTs. Among the characteristics tested, plant height (i.e., stem length) and weight of 100 seeds were collected in both Japan and the US by each developer, and thus were chosen as representative data to assess for similarity of results. US CFT data for these agronomic traits were taken from publicly available petition documents for each event (USDA-APHIS 2018). As shown in Fig. 2, for both characteristics, individual data points corresponding to each pair of mean values for GM soybean and non-GM comparator tested in US CFTs distributed around Y = X as did values from Japan CFTs, and strong correlation was observed (R ≥ 0.90). A narrower range of values was observed in the US dataset (Fig. 2) likely because the data is based on CFTs in multiple sites. Consistency in the comparability between GM soybean and non-GM control in these two agronomic endpoints obtained in CFTs conducted in both Japan and US irrespective of traits conferred by the inserted genes supports that the dataset collected in US is transportable and can be utilized in the for ERA in Japan.

Based on a high level of familiarity (familiarity of the crop, familiarity with GM soybeans, and familiarity based on existing agronomic data), we propose that the scope of the current regulatory framework in Japan, where GM maize events with certain “familiar genes” are exempt from the CFTs in Japan and the cultivation approval considers field data obtained in other countries, is expanded to include GM soybeans.

The exemption rule for GM maize was adopted in 2014, and was based on a high-level familiarity and accumulated experience conducting ERAs for GM maize. In the case of GM maize, it was determined that the agronomic data collected from field trials in the US is not different from the agronomic data collected from in-country CFTs. The field data of a GM maize obtained in other countries was determined to be sufficient to inform the ERA in Japan if: (1) the mode of action of the protein derived from the inserted gene is clearly defined from the scientific point of view; and (2) the effect of the inserted gene is comparable to that of the other traits which have already been approved (MAFF 2014). It is worth noting that the scope of the exemption of CFTs in Japan has recently been expanded to include GM cotton in 2019 which is also based on accumulated experience conducting ERAs for GM cotton in Japan.

## Conclusion

In this paper, we have summarized and compared agronomic characteristics for 11 soybean events assessed in CFTs in Japan. This data helps build familiarity for the crop, by providing agronomic data which was not previously publicly disclosed. Also, this comprehensive dataset establishes a baseline of agronomic data which can be used to assess biological relevance of agronomic differences. When CFTs in Japan are required for the events with novel genes or traits, the familiarity of non-GM soybeans in CFTs in Japan that have been summarized in this paper will provide the range of natural variation in agronomic characteristics to better evaluate biological relevance. Furthermore, by comparing the agronomic data from CFTs in Japan and the US, we have demonstrated that the agronomic data collected in multi-location field trials in other countries can be used to inform the ERA for Japan. The concept where risk assessors in one country can use the data and assessments from other countries to inform their own assessments is known as data transportability (Ahmad et al. 2016; Garcia-Alonso et al. 2014; Nakai et al. 2015). As part of problem formulation and leveraging a science-based approach, existing data should be used to inform the risk assessment and can help determine when a CFT in Japan is needed to assess risk. Therefore, we propose that the scope of the current regulatory framework in Japan, where GM maize events with certain “familiar genes” are exempt from the CFTs in Japan and the cultivation approval considers field data obtained in other countries, is expanded to include GM soybeans. These familiarity-based approaches to the ERAs of GM soybean which will have potential to reduce the regulatory burden for both developers and regulatory authorities, while still ensuring the quality of risk assessment.

More than two decades have passed since the regulation and related risk assessment processes of GM crop were initially developed. The assessment frameworks in Japan and other countries have evolved based on our experience and increasing familiarity with GM crops and traits over time. The concept of familiarity and the comparative assessment, which was originally proposed long ago, has been providing the foundation for effective and harmonized ERA. Extensive discussion regarding how to make use of data that is globally relevant from ERAs and CFTs to increase familiarity is encouraged for more scientifically sound and risk-proportionate ERA.

## Electronic supplementary material

Below is the link to the electronic supplementary material.

Supplementary material 1 (PDF 210 kb)

Supplementary material 2 (XLSX 23 kb)
