# Supplementary material for: Consideration of familiarity accumulated in the confined field trials for environmental risk assessment of genetically modified soybean (Glycine max) in Japan
Source: Transgenic Res. 2020 Jan 29;29(2):229–42. doi: 10.1007/s11248-020-00193-z (PMC7067755; doi:10.1007/s11248-020-00193-z)
Supplement: Supplementary file 1 — Supplementary material 1 (PDF 210 kb) [file 11248_2020_193_MOESM1_ESM.pdf]

Title: Consideration of Familiarity Accumulated in the Confined Field Trials for Environmental Risk Assessment of Genetically Modified Soybean (*Glycine max*) in Japan

Journal: Transgenic Research

Author information:

Akane Matsushita[a], Hidetoshi Goto[b], Yasuyuki Takahashi[c], Mai Tsuda[d,e], Ryo Ohsawa[d,e]

[a] Dupont Production Agriscience K.K. Sanno Park Tower, 2-11-1, Nagata-cho, Chiyoda-ku, Tokyo 100-6111 Japan

[b] Bayer CropScience, Kyobashi Soseikan Building 6F, 2-5-18, Kyobashi, Chuo-ku, Tokyo, 104-0031 Japan

[c] Dow AgroSciences Japan Ltd. Sanno Park Tower, 2-11-1, Nagata-cho, Chiyoda-ku, Tokyo 100-6111 Japan

[d] Gene Research Center, Tsukuba Plant Innovation Research Center, University of Tsukuba, 1-1-1 Tennodai, Tsukuba, Ibaraki 305-8572 Japan.

[e] Faculty of Life and Environmental Sciences, University of Tsukuba, 1-1-1 Tennodai, Tsukuba, Ibaraki 305-8572 Japan.

*Corresponding Author

E-mail: osawa.ryo.gt@u.tsukuba.ac.jp

Phone: +81-29-853-6674

Content:

Online Resource 1, 3, 4, 5 and 6.

Online Resource 1. The dates and the sites of the eleven CFTs of GM soybeans used in this study

OECD UI	Period	Site
DAS-68416-4	2012, Jul. - 2013, Jan.	Fukuoka
DAS-444Ø6-6	2012, Jul. - 2013, Jan.	Fukuoka
DAS-81419-2	2013, Aug. - 2014, Feb.	Fukuoka
DP-356Ø43-5	2005, Jul. - 2005, Dec.	Shizuoka
DP-3Ø5423-1	2007, Jun. - 2007, Dec.	Tochigi
MON-89788-1	2006, May - 2006, Nov.	Ibaraki
MON-87769-7	2008, Jul. - 2009, Jan.	Ibaraki
MON-877Ø1-2	2009, Jul. - 2010, Jan.	Ibaraki
MON-877Ø5-6	2009, Jul. - 2010, Jan.	Ibaraki
MON-877Ø8-9	2010, Jun. - 2011, Jan.	Ibaraki
MON-87751-7	2014, Jun. - 2015, Jan.	Ibaraki

Online Resource 3. Common agronomic characteristics evaluated across developers

Measurement endpoint	Definition
Germination rate	Ratio of the number of germinated seeds to the number of seeds planted
Flowering stage	Date by which 50% of plants flowered, or the dates of the beginning and the end of flowering
Maturity stage	Date by which 80% to 100% of plants reached senescence
Main stem length	Distance from the cotyledon node to the apical meristem of the main stem
Number of nodes	Number of nodes on the main stem per plant
Number of branches	Number of branches from the main stem per plant
Number of pods	Number of grain-carrying pods per plant
Pod shattering	Ease of shattering of pods
Weight of grain	Mass of all harvested grain per plant
Weight of 100 grains	Mass of 100 harvested grains

Online Resource 4. Viability (average staining %) and size (mean diameter; µm) of pollen from GM and non-GM NIL (control) soybeans

OECD UI	Staining (%)[a]		Diameter (µm)[a]	
	GM	Control	GM	Control
DAS-68416-4	98.7	99.2	26.5	26.6
DAS-444Ø6-6	99.5	99	26	26
DAS-81419-2	95.5	95.5	24.9	24.8
DP-356Ø43-5	98	99	14.6	15.1
DP-3Ø5423-1	98	98	25.8	26
MON-89788-1	NC[b]	NC	NC	NC
MON-87769-7	NC	NC	NC	NC
MON-877Ø1-2	NC	NC	NC	NC
MON-877Ø5-6	NC	NC	NC	NC
MON-877Ø8-9	NC	NC	NC	NC
MON-87751-7	99.0	99.1	25.5	25.4

[a]Average values for each measurement endpoint are provided however, since each value was copied from original dossiers without modification, the number of significant figures are not consistent across studies.

[b]NC: Not conducted. For these events, viability and size of pollen was analyzed by qualitative visual observation, and no quantitative data was provided.

Online Resource 5. Production of harmful substance in GM soybean evaluated by soil tests

Event Name	Succeeding crop test[a]				Plow-in test[a]				Soil microflora test[a]					
	G/SE rate (%)		dry weight (g)		G/SE rate (%)		dry weight (g)		bacteria ($x10^{6}$)		filamentous fungi ($x10^{4}$)		actinomycetes ($x10^{4}$)	
	GM	Control	GM	Control	GM	Control	GM	Control	GM	Control	GM	Control	GM	Control
DAS-68416-4	97	93	0.13	0.14	99	97	0.2	0.2	30	38	6.3	7.8	310	340
DAS-444Ø6-6	95	94	0.17	0.17	100	98	0.17	0.18	49	51	7.6	8.1	360	340
DAS-81419-2	100	99	0.11	0.12	100	99	0.24	0.25	19	15	7.2	6	270	320
DP-356Ø43-5	98	99	0.17	0.17	98	98	0.17	0.17	1.29	2.85	9.2	29.3	32.8	15
DP-3Ø5423-1	100	99	0.043	0.043	97	98	0.053	0.053	4.97	4.67	31.8	26.8	112	114
MON-89788-1	94.02	93.16	13.86	13.66	99.15	97.44	14.14	14.04	2300	2100	1900	2200	10300	4600
MON-87769-7	98.29	97.44	0.62	0.69	99.13	98.30	1.01	1.21	300	330	230	240	3600	2800
MON-877Ø1-2	95	97.5	0.87	0.80	97.5	98.3	0.87	0.88	1600	170	600	700	12000	12000
MON-877Ø5-6	98.3	100	1.05	1.08	99.2	98.3	1.02	1.05	710	870	580	560	8000	11000
MON-877Ø8-9	97.5	99.2	0.56	0.57	92.5	95.8	0.70	0.67	730	760	2600	3900	8000	7900
MON-87751-7	92.2	95.8	1.0	1.1	90.0	93.3	1.1	1.0	56	58	25	25	4500	4800

[a]Average values for each measurement endpoint are provided. Since each value was taken from original dossiers without modification, significant figures reported are not consistent.

Online Resource 6. Outcross rate of GM soybean

Event Name	Outcross rate (%)<br>[progeny positive for trait/total progeny measured]
DAS-68416-4	0.16 [5/3129]
DAS-444Ø6-6	0.23 [7/3080]
DAS-81419-2	0.10 [3/2876]
DP-356Ø43-5	0 [0/432]
DP-3Ø5423-1	0 [0/1600]
MON-89788-1	0 [0/500]
MON-87769-7	0 [0/491]
MON-877Ø1-2	0 [0/480]
MON-877Ø5-6	0 [0/480]
MON-877Ø8-9	0 [0/500]
MON-87751-7	0 [0/480]
